# Unravelling Taxonomic Complexity in Elusive Cetaceans: Mitogenome Insights into Evolutionary History and Cryptic Diversity of Bryde's Whales

**DOI:** 10.1002/ece3.73614

**Published:** 2026-05-03

**Authors:** Dominique Paynee, Paulette Bloomer, Gwenith Penry, Simon Elwen, Ada Natoli, Vaisakh Dhanalakshmy, Thyago Cardoso, Els Vermeulen

**Affiliations:** ^1^ Mammal Research Institute Whale Unit, Department of Zoology and Entomology University of Pretoria Pretoria South Africa; ^2^ Molecular Ecology and Evolution Programme, Department of Biochemistry, Genetics and Microbiology University of Pretoria Pretoria South Africa; ^3^ Institute for Coastal and Marine Research Nelson Mandela University Gqeberha South Africa; ^4^ Sea Search, Research and Conservation, Muizenberg, South Africa Sea Search, Research and Conservation Muizenberg South Africa; ^5^ Department of Botany and Zoology Stellenbosch University Stellenbosch South Africa; ^6^ UAE Dolphin Project Initiative, Environmental Science and Sustainability Department, College of Natural and Health Science Zayed University Dubai UAE; ^7^ M42 Health, M42 Environmental Sciences, Omics Centre of Excellence M42 Health Abu Dhabi UAE

**Keywords:** baleen whale, conservation unit, cryptic diversity, mitogenome, phylogenomics, taxonomy

## Abstract

Cetaceans, despite their ability to cross wide distances, enabling mostly high levels of gene flow, can develop unique genetic lineages driven by variations in life‐history traits, diverse migratory routes and local adaptation, complicating taxonomy and delineation of conservation units. Comprehensive genetic data are essential to overcome the challenges of obtaining suitable sample sizes from wild cetaceans while untangling cryptic species diversity. The Bryde's whale ( *Balaenoptera edeni* ) has a complicated discovery and taxonomic history that hinders accurate conservation assessments. In southern Africa, two populations are recognised: the inshore (SAi) and the offshore southeast Atlantic population (SEA). This study examines their phylogenetic relationships and evolutionary history relative to global populations and other balaenopterids. Complete mitochondrial genomes from SAi and SEA individuals were analysed alongside mitogenomes from the Arabian/Persian Gulf (United Arab Emirates, UAE, this study), North Pacific offshore ecotypes (GenBank), coastal Japan's inshore ecotype (GenBank) and five additional balaenopterid species (GenBank). Phylogenomic analyses revealed that each population forms a distinct lineage. SAi and SEA individuals grouped with the subspecies *B. edeni brydei* (offshore ecotype), despite the SAi occurring inshore, whereas the UAE individual was more closely related to *B. edeni edeni* and *B. ricei*. Divergence dating estimates suggest that the southern African, UAE and North Pacific populations shared a most recent common ancestor approximately 4–5 million years ago. These findings emphasise the need to reassess conservation units, population estimates and potentially reclassify new subspecies or species among Bryde's whales. This study demonstrates how mitogenome data can detect cryptic diversity in elusive cetaceans, directing more effective conservation of unique genetic lineages.

## Graphical abstract

The Bryde's whale is one of the least understood baleen whale species globally, limiting accuracy of regional and global conservation assessment. This study shows the occurrence of unique genetic lineages and evolutionary histories among Bryde's whale populations. The strong evidence of cryptic diversity within the species is a key finding in delineation of conservation units through the species distribution aiding in effective preservation of genetic diversity.

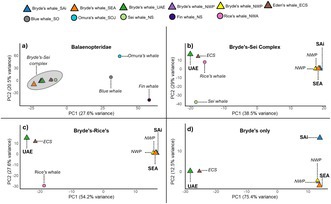

## 1 Introduction

Cetaceans exhibit a range of life history traits including complex social structures, relatively long lifespans, long generation times, migratory behaviour and potentially wide home ranges that collectively complicate the process of defining species and population boundaries (Vachon et al. 2018). This, combined with the challenges of collecting suitable data, limits taxonomic resolution and effective protection of wild populations (Taylor, Archer, et al. 2017; Taylor, Perrin, et al. 2017). Effective conservation depends on clearly defined conservation units within species, accurate estimates of population size and gene flow, and an understanding of populations' ability to persist and adapt in response to environmental changes (Hohenlohe et al. 2021). Cetaceans present particular challenges in this regard as they are one of the most complex taxonomical groups of marine mammals and have been described as ‘under‐classified’ in terms of the number of recognised species and subspecies (Taylor, Perrin, et al. 2017). Their often wide distribution ranges make it difficult to sample and observe individuals and populations (Morin et al. 2023; Taylor, Perrin, et al. 2017; Taylor, Archer, et al. 2017), resulting in many species with unresolved or complex taxonomies. Furthermore, some populations inhabit remote or inaccessible areas, increasing the challenges to studying them while allowing for the development of distinct populations that may warrant recognition as subspecies or even separate species (Taylor, Archer, et al. 2017). Characteristics such as a marine habitat (limiting accessibility of observation and study) and large body size (restricting housing of skeletal evidence) have historically complicated the use of morphology‐based approaches to species and subspecies delineation in cetaceans (Morin et al. 2023). To overcome these challenges, the collection of tissue samples through biopsy procedures of free‐ranging animals or sampling of stranded individuals for use in alternative methods, particularly those leveraging genomic data, have been implemented. Enhancing our understanding of population differentiation and taxonomic boundaries within species using genomics provides essential information for refining management strategies such as mitigating anthropogenic threats, defining protected areas and optimally timing conservation interventions (Taylor, Archer, et al. 2017).

The Bryde's whale ( *Balaenoptera edeni* ) is a prominent example of a cetacean taxon with an unresolved and complex taxonomy, largely stemming from a convoluted history of discovery (Constantine et al. 2018; Penry et al. 2018). Combined with their notoriously elusive behaviour, this species is a prime example of a cetacean for which sampling is limited and knowledge is biased toward a few regions across its range. The species was first described by Anderson (1878) and named Eden's whale ( *B. edeni* ) from a stranded individual in Burma (now Myanmar). This initial description was then followed by Olsen's (1913) description of a ‘new baleen whale’ off the South African coast, which was assigned the name Bryde's whale ( *B. brydei* ). Subsequent taxonomic examination synonymised Eden's and Bryde's whales as a single species (Junge 1950) based on skeletal evidence only, leading to decades of confusion around accurate species listing for these phenotypically similar baleen whales. The uncertainty persists today due to the absence of a type specimen for *B. brydei* (Olsen 1913) and the lack of genetic verification for the holotype of *B. edeni* (Anderson 1878; Constantine et al. 2018; Penry et al. 2018). Currently, Bryde's whales are considered part of the ‘Bryde's‐sei complex’, which includes sei whales ( *B. borealis* ) as well as the most recently recognised species, Rice's whale (Rosel et al. 2022). This complex, comprising Bryde's whales and ‘Bryde's‐like’ species, is frequently referenced and used in phylogenetic analyses while taxonomic uncertainties are being resolved.

Two populations critical to resolving the taxonomy of Bryde's whales occur around the southern African region: the southeast Atlantic offshore population (SEA) and the South African inshore population (SAi). Following Olsen's (1913) initial description of *B. brydei* , later research identified two allopatric ecotypes in the region: an inshore ecotype (SAi) and an offshore ecotype (SEA) (Best 1977, 2001). These two ecotypes are separated based on differences in prey, morphology, geographical distribution, and ecology (Best 1977, 2001). Individuals of the SEA inhabit the south west coast of Africa, migrating between South Africa in summer months and Gabon in winter months, whereas the SAi is non‐migratory and has a restricted distribution over the continental shelf of South Africa, primarily on the south and east coasts (Best 2001). Morphologically, SEA individuals are larger than SAi individuals and typically have numerous oval‐shaped scars on their bodies from the bites of cookie‐cutter sharks ( *Isistius brasiliensis* ), correlating with their offshore distribution (Best 1977, 2001). Furthermore, distinct differences in prey species and reproductive biology (including size at sexual maturity, reproductive cycles and size at birth) supported their recognition as two allopatric ecotypes in southern Africa (Best 1977, 2001). The clear description of the two ecotypes by Best (1977) revealed that Olsen's initial description of *B. brydei* included features of both SAi and SEA individuals (Constantine et al. 2018), contributing to the currently unclear taxonomy. Nonetheless, there are other documented cases of inshore and offshore ecotypes, such as off the coast of Japan (Sasaki et al. 2005) and in the Northern Indian Ocean (Kershaw et al. 2013), which has directed the current nomenclature and listing of two subspecies of Bryde's whale: a larger pelagic form ( *B. edeni brydei* , Bryde's whale) and a small coastal form ( *B. edeni edeni* , Eden's whale) (Committee on Taxonomy 2025).

A previous study using mitochondrial control region data concluded that the SEA and SAi populations are distinct at least at the subspecies level (Penry et al. 2018). More recent analyses suggested historical links between SAi and the Eastern Indian Ocean (EIO), as well as a low number of divergent haplogroups across both SAi and SEA regions (Paynee et al. 2026). Although only a few new mitogenomes were generated in this study, they captured the limited haplotype diversity previously reported for SAi and SEA (Penry et al. 2018). Globally, mtDNA control region studies indicate multiple distinct Bryde's whale lineages from different ocean regions (Kanda et al. 2007; Kershaw et al. 2013; Luksenburg et al. 2015; Penry et al. 2018; Dalpaz et al. 2023). However, the limited variability within the control region in some taxa, coupled with incomplete lineage sorting (ILS), can limit its power to delineate subspecies or species boundaries (Morin et al. 2023). To strengthen these initial taxonomic inferences (Penry et al. 2018) and improve understanding of evolutionary and phylogenetic relationships (Paynee et al. 2026), broader geographic comparisons are needed in combination with more robust, comprehensive genetic datasets, such as mitochondrial genomes (mitogenomes) (Morin et al. 2023). Morin et al. (2023) demonstrate that the mitogenome is a proven and reliable tool for species and subspecies identification, offering high diagnosability through clear divergence tests, and it is increasingly applied across cetacean taxa to evaluate subspecies classification and population boundaries (e.g., Archer et al. 2019; Leslie et al. 2019; Van Cise et al. 2019; Bachmann et al. 2021).

In this study, we aimed to refine and expand the current understanding of the broader phylogenetic relationships, evolutionary history and divergence patterns of the southern African Bryde's whale populations. We generated new mitogenome data and analysed these in combination with representative Bryde's whale individuals from the Indian and North Pacific Oceans, respectively as well as additional balaenopterid species.

## 2 Materials and Methods

### 2.1 Sample Collection and DNA Extraction

**FIGURE 1:**
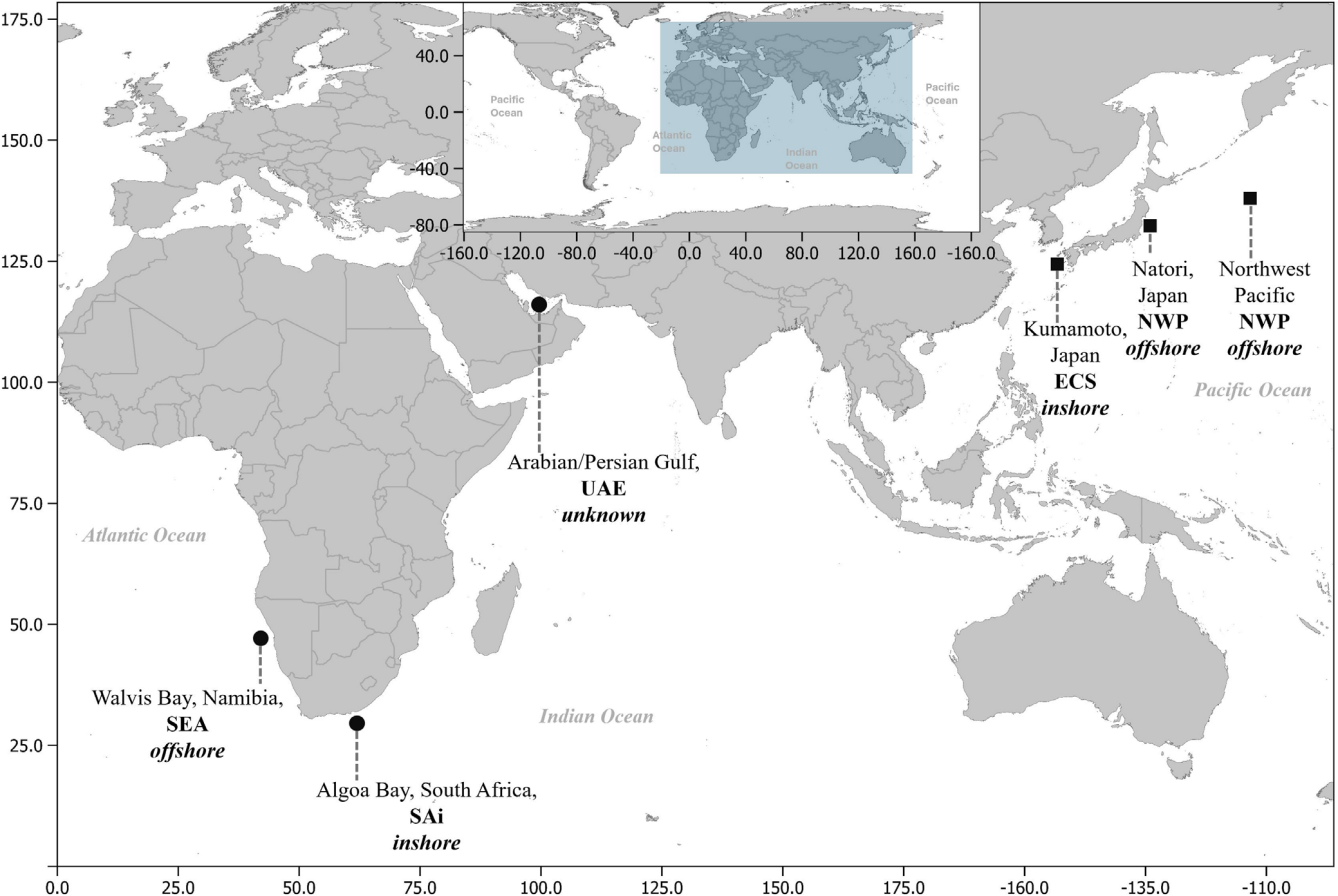
Sampling locations of Bryde's whales (dots) used for de novo mitogenome assembly and GenBank‐sourced mitogenomes of Bryde's whale and Eden's whale (squares). The ecotype classification of each individual is indicated as ‘inshore’ or ‘offshore’.

One biopsy sample and two necropsy samples from strandings were used to generate three mitogenomes of Bryde's whales from different ocean regions. The samples originated from coastal waters on the east coast of South Africa (southwestern Indian Ocean), the southeast Atlantic off Namibia, and the Arabian/Persian Gulf off the coast of the United Arab Emirates (UAE) (Figure 1). These correspond to populations herein referred to as the South African inshore (SAi), southeast Atlantic (SEA) and UAE populations. The SAi sample was collected from a live female whale in Algoa Bay, South Africa, in 2023 (Figure 1). The tissue sample of the SEA individual was from a stranding in Walvis Bay, Namibia in 2012 (Penry et al. 2018), and the third sample from a stranding on the coastline of Dubai (UAE). Prior to extraction, skin samples were frozen at −20°C. Thereafter DNA was extracted from the SAi and SEA individuals in the University of Pretoria Genomics Laboratory, where standard decontamination procedures were followed, including sterilisation of surfaces and equipment using UV light treatment, 10% bleach and 70% ethanol. DNA extractions from SAi and SEA samples were conducted using the NucleoBond HMW DNA kit (Macherey‐Nagel, South Africa), according to the manufacturer's protocol. The skin samples were digested overnight rather than the suggested 30 min to maximise cell lysis.

The UAE specimen was in an advanced state of decomposition; therefore, two separate DNA extraction procedures were completed in a laboratory onboard the OceanX research vessel using the QIAGEN MagAttract HMW DNA Kit (Set‐II) (Qiagen, Germany), and re‐extraction using the QIAGEN G2 buffer method (Qiagen, Germany) followed by Genomic‐tip purification. Standard laboratory procedures at the University of Pretoria Genomics Laboratory were followed by the laboratory onboard the OceanX research vessel.

Additional mitogenomic data were sourced from GenBank entries, which included different species of balaenopterids and other Bryde's whales from different ocean regions (Figure 1, Table 1). Bryde's whale entries representing Japan's offshore population ( *B. edeni brydei* ) (NC 006928.1 & AB201259.1) are referred to using the population code NWP, while Eden's whale ( *B. edeni edeni* ) (NC 007938.1), representing Japan's inshore population in the East China Sea, is referred to as ECS.

**TABLE 1:** Bryde's whale de novo mitogenomes and balaenopterid mitogenomes from GenBank included in this study, showing species name, accession numbers, sampling location and ocean region.

Common name (study)	Species name	GenBank accession number	Sampling location, ocean region	Population/Region acronym
Bryde's whale (this study)	*Balaenoptera edeni*	PZ143434	Algoa Bay, South Africa Southern Indian Ocean	*SAi*
Bryde's whale (this study)	*Balaenoptera edeni*	PZ143435	Walvis Bay, Namibia Southeast Atlantic Ocean	*SEA*
Bryde's whale (this study)	*Balaenoptera edeni*	PZ143436	Arabian/Persian Gulf, UAE Northern Indian Ocean	*UAE*
Bryde's whale (Sasaki et al. 2005)	*Balaenoptera edeni brydei*	NC 006928.1	Northwest Pacific Ocean	*NWP*
Bryde's whale (Sasaki et al. 2005)	*Balaenoptera edeni brydei*	AB201259.1	Natori, Japan, Northwest Pacific Ocean	*NWP*
Eden's whale (Sasaki et al. 2005)	*Balaenoptera edeni edeni*	NC 007938.1	Kumamoto, Japan, Northwest Pacific Ocean	East China Sea (*ECS*)
Rice's whale (Rosel et al. 2021)	*Balaenoptera ricei*	PQ997938.1	Gulf of Mexico, USA, Northwest Atlantic Ocean	*NWA*
Omura's whale (Sasaki et al. 2006)	*Balaenoptera omurai*	NC 007937.1	Tsunoshima Island, Japan, Northwest Pacific Ocean	Sea of Japan (*SOJ*)
Sei whale	*Balaenoptera borealis*	OZ239556.1	Vaul, United Kingdom, North Sea	*NS*
Fin whale	*Balaenoptera physalus*	OZ239533.1	Burray, United Kingdom, North Sea	*NS*
Blue whale	*Balaenoptera musculus*	ON675436.1	Southern Ocean	*SO*

### 2.2 Library Preparation and Oxford‐Nanopore Technologies (ONT) Sequencing

Libraries were prepared for the SAi and SEA samples using 1 μg of high‐molecular‐weight DNA obtained from DNA extraction. These long‐read libraries were prepared using the Ligation Sequencing Kit V14 (SQK‐LSK114; Inqaba Biotec, Pretoria). Due to lower initial DNA integrity of the UAE sample (compared to SAi and SEA), 3 μg of high‐molecular‐weight DNA was used to construct libraries using the Ligation Sequencing Kit (SQK‐LSK114, Oxford Nanopore Technologies, UK). After sequencing runs, adapter trimming and base calling were performed on all samples using the PromethION sequencing software, MiKNOW v 24.11.10 and Dorado v 7.2.3. Following library preparation, sequencing was performed using R10.4.1 flow cells (FLO‐PRO114M; Oxford Nanopore Technologies) on the PromethION platform.

### 2.3 Reference Assisted De Novo Mitogenome Assembly

The FASTQ files containing long Oxford‐Nanopore Technologies (ONT) reads for every individual underwent a quality check (maintaining reads of Q > 15) and trimming using NanoPlot v 1.44.1 (De Coster and Rademakers 2023) and Nanofilt v 2.8.0 (De Coster and Rademakers 2023), respectively. Thereafter, the reads were mapped to a reference mitogenome of a Bryde's whale ( *Balaenoptera brydei* , NC 006928.1) using minimap2 v 2.28 (Li 2018) to extract all mitochondrial reads. The mean coverage depth were calculated in SAMtools v 1.20 (Li et al. 2009) utilising the ‘samtools coverage’ tool. Additional filtering steps included removal of reads smaller than 1 kb using BLAST v 2.13.0 (Camacho et al. 2009) to match the contigs with the reference assembly and account for potential nuclear read contamination. Each mitogenome was assembled using FLYE v 2.9.5 (Kolmogorov et al. 2019) with three rounds of internal iterations followed by one more polishing round using Racon v 1.5.0 (Vaser et al. 2017). The mitogenome assemblies were assessed for completeness using QUAST v 5.3.0 (Gurevich et al. 2013) after each polishing step. The length of contigs in each assembly was assessed and the largest contig, spanning approximately 16,400 bp (the size of the cetacean mitogenome), was extracted, aligned to the reference genome using MAFFT v 7 (Katoh et al. 2019) and trimmed in MEGA11 v 11.0.13 (Tamura et al. 2021). All mitogenomes were annotated in MITOS2 (Donath et al. 2019) using the galaxy platform.

### 2.4 Single Nucleotide Polymorphisms (SNPs) and Divergence Analyses

Four datasets were created for analyses (Table 2), all containing the three de novo Bryde's whale mitogenomes and different combinations of divergent balaenopterid species. These datasets were curated hierarchically to assess how the inclusion of different species changed the overall patterns of divergence between Bryde's whale individuals. Divergence levels between Bryde's whale individuals and all other species were investigated by measure of the number of Single Nucleotide Polymorphisms (SNPs) differences between individuals using PLINK v 2.3 (Chang et al. 2015) and then by generating Principal Components Analyses (PCA) plots in R v 4.4.3 (RCore team 2025).

**TABLE 2:** Four different mitogenome datasets curated for analyses containing different combinations of balaenopterid species.

Dataset name	Species included
Balaenopteridae	Six Bryde's whales (incl. one Eden's whale), and five balaenopterid species (blue whale, sei whale, Omura's whale, fin whale, Rice's whale)
Bryde's‐sei complex	Six Bryde's whales (incl. one Eden's whale), and two ‘Bryde's like’ whales (one Rice's and one sei whale)
Bryde's‐Rice's	Six Bryde's whales (incl. one Eden's whale), and one Rice's whale
Bryde's only	Six Bryde's whales (incl. one Eden's whale)

### 2.5 Phylogenetic Analyses

Maximum likelihood trees were generated for the ‘Balaenopteridae’ dataset using IQ‐Tree2 (Minh et al. 2020), running 10,000 bootstrap replicates to assess node support. Model tester flags were incorporated in IQ‐Tree2 to discern a best fit model for the dataset. The outputs of IQ‐Tree2 were then visualised on iTOL (Interactive Tree of Life) v 1.0 (Letunic and Bork 2021). In addition, phylogenetic relationships using Bayesian inference were computed on the Balaenopteridae dataset using MrBayes (1 million MCMCs, sampling frequency = 500, burnin = 25%, number of runs = 2, GTR + I + G model) (Ronquist et al. 2012) as confirmation of the relationships inferred in IQ‐Tree2. A time‐calibrated phylogenetic tree was then generated for the ‘Bryde's‐sei complex’ dataset using BEAST2 v 2.6.6 (Bouckaert et al. 2014) to estimate possible divergence times between Bryde's whale populations using the sei whale as an outgroup. The TN93 + G substitution model was selected aligning with the most suitable model dictated in IQ‐Tree2 model testing. These data were then modelled using a strict clock and an estimated substitution rate of 0.3% bp^−1^ My^−1^ as found previously for baleen whales (Jackson et al. 2009). A Yule speciation model was used for generation of this tree, as analyses included two divergent species (Rice's and sei whale). The model was run with 10 million Markov Chain Monte Carlo (MCMC) iterations, storing every 1000th sample for calculation of posterior probability. Convergence and ESS values were verified in Tracer v 1.7.2 (Rambaut et al. 2018) ensuring all ESS values were > 200. The tree outputs of BEAST were then run on TreeAnnotator v 2.6.6 (Helfrich et al. 2018) to generate a maximum clade credibility tree, which was visualised in Figtree v 1.4.4 (Rambaut 2018).

## 3 Results

### 3.1 Reference Assisted De Novo Mitogenome Assembly

The full mitogenome (~16,400 bp) for three Bryde's whale individuals (SAi, SEA, and UAE) was extracted and successfully assembled from ONT reads. Genome completeness tests indicated that each mitogenome assembly spanned 100% of the Bryde's whale GenBank mitogenome sequence (NC 006928.1) which was used as a reference (Sasaki et al. 2005). The average read error rate after filtering ranged from 2.82% (UAE) to 2.09% (SAi), with the SEA sample estimated at 3.47%. Although these rates were high (as expected for ONT data) it was offset by mean depth of coverage for each sample, increasing accuracy. The mean depth of coverage was 12,668× for SAi, 1676× for SEA and 12,245× for UAE. The average GC content for each mitogenome was within an expected range for mammals (40%–42%) (Romiguier et al. 2010). The annotation of each mitogenome resulted in 13 protein‐coding genes, 22 tRNAs and 2 rRNAs, and the mitochondrial control region as expected for vertebrate mitochondrial genomes.

### 3.2 Intra‐ and Interspecies Divergence

Different numbers of SNPs were detected across each of the four mitogenome datasets analysed, with an increasing number of SNPs detected when more divergent species were included. No obvious clustering of SNPs was visually apparent, and variation appeared broadly distributed across the mitogenome. Within the largest and most expansive dataset (Balaenopteridae), 2831 SNPs were detected among the six different balaenopterid species. In the Bryde's‐sei complex dataset, 967 SNPs were detected. When only the Rice's whale and Bryde's whales were combined (Bryde's‐Rice's), 742 SNPs were detected, and within Bryde's whales alone (including one Eden's whale), 579 SNPs were detected. Between the two NWP individuals, 27 SNPs were detected, providing a ‘threshold’ for the number of differences between individuals from the same region and population.

**FIGURE 2:**
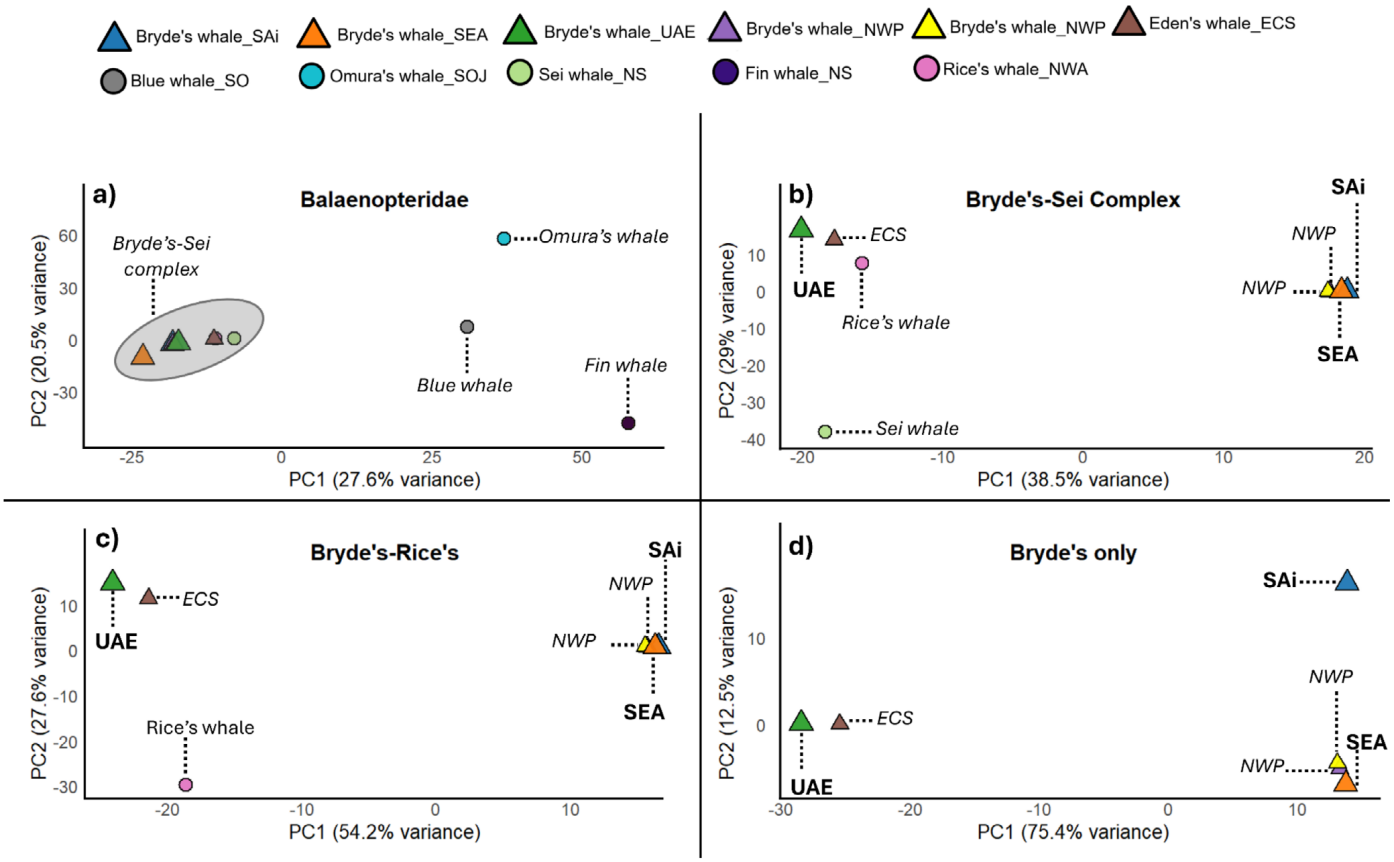
Principal Components Analyses (PCA) of four different mitogenome datasets based on number of SNP differences. Bryde's and Eden's whale individuals are indicated by triangles and other species by circles. (a) Mitogenomes of five balaenopterid species and six Bryde's (incl. one Eden's) individuals, (b) Mitogenomes of six Bryde's, (incl. one Eden's) and two ‘Bryde's‐like’ whale (Rice's and sei) individuals, (c) Mitogenomes of six Bryde's whale (incl. one Eden's) and one Rice's whale individual, (d) Mitogenomes of six Bryde's whales (incl. one Eden's whale).

The patterns of divergence between individuals from different Bryde's whale populations shifted depending on the inclusion of different divergent species (Figure 2). Figure 2a shows the divergence pattern between five different balaenopterid species and six Bryde's whales (incl. one Eden's whale). Overall, the Bryde's and Eden's whales clearly separate from all other species and cluster closest to the species that are included in the species complex, namely Rice's and sei whales. The SAi and UAE individuals clustered very closely with the two NWP Bryde's whale individuals, whereas the SEA individual is more distant from this cluster (Figure 2a). Once the more divergent species were removed from the dataset, the UAE individual consistently clustered with the ECS individual (Eden's whale) (Figure 2b–d). In contrast to this, the SAi and SEA individuals consistently formed clusters with the NWP individuals. However, when comparing only Bryde's whale mitogenome differences, the SAi individual clearly separated from the SEA and NWP individuals (Figure 2d).

### 3.3 Phylogenetic Relationships

**FIGURE 3:**
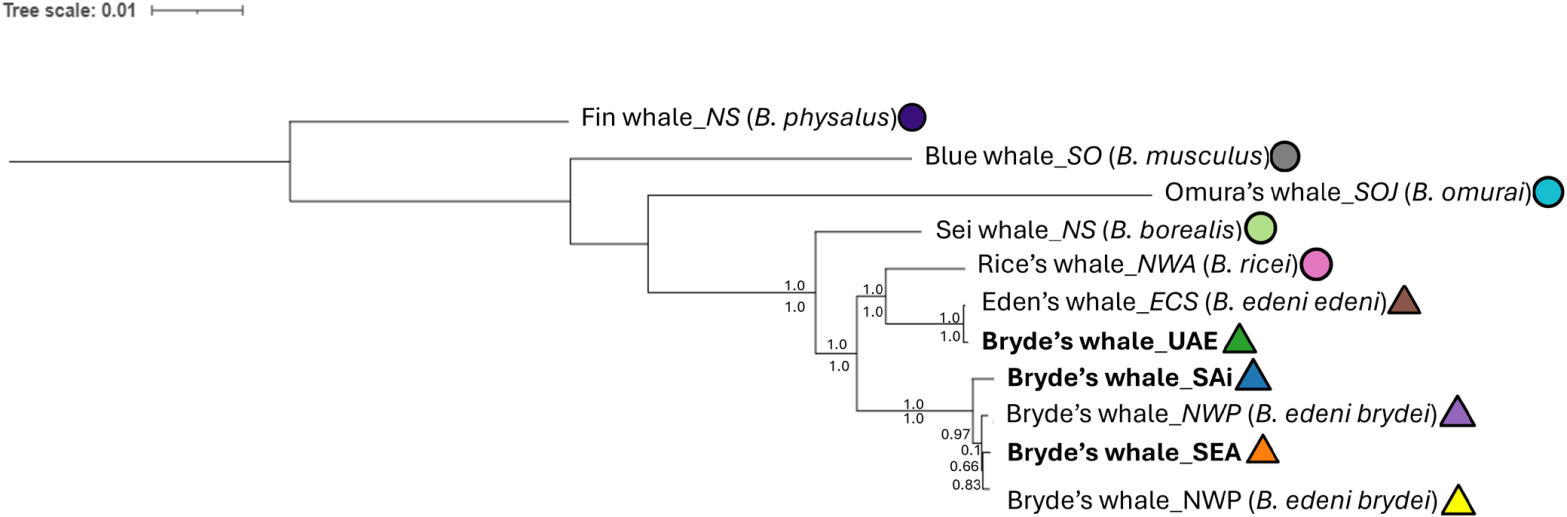
Maximum likelihood tree based on a most suitable model of evolution for the data between mitogenomes of balaenopterid species with 10,000 bootstrap replicates. The maximum likelihood bootstrap support is indicated above the branch and Bayesian Posterior Probability (BPP) values inferred by MrBayes indicated below.

The inferred phylogenetic relationships supported the patterns of divergence observed between Bryde's whale individuals from different populations. When compared with individuals of the broader balaenopterid dataset, the representative NWP individuals formed a cluster with the SAi and SEA individuals, whereas the UAE individual clustered as a sister taxon to the ECS lineage (Figure 3). However, there was low nodal support for the placement of the NWP individuals relative to the SEA. Although the southern African individuals clustered together, the placement of SEA on a separate branch indicated the genetic differentiation from SAi. The larger cluster (containing SAi, SEA and NWP) formed a distantly related sister group to the ECS, UAE and Rice's whale grouping (Figure 3). Phylogenetic relationships based on maximum likelihood and Bayesian inference were congruent (Figure S1).

**FIGURE 4:**
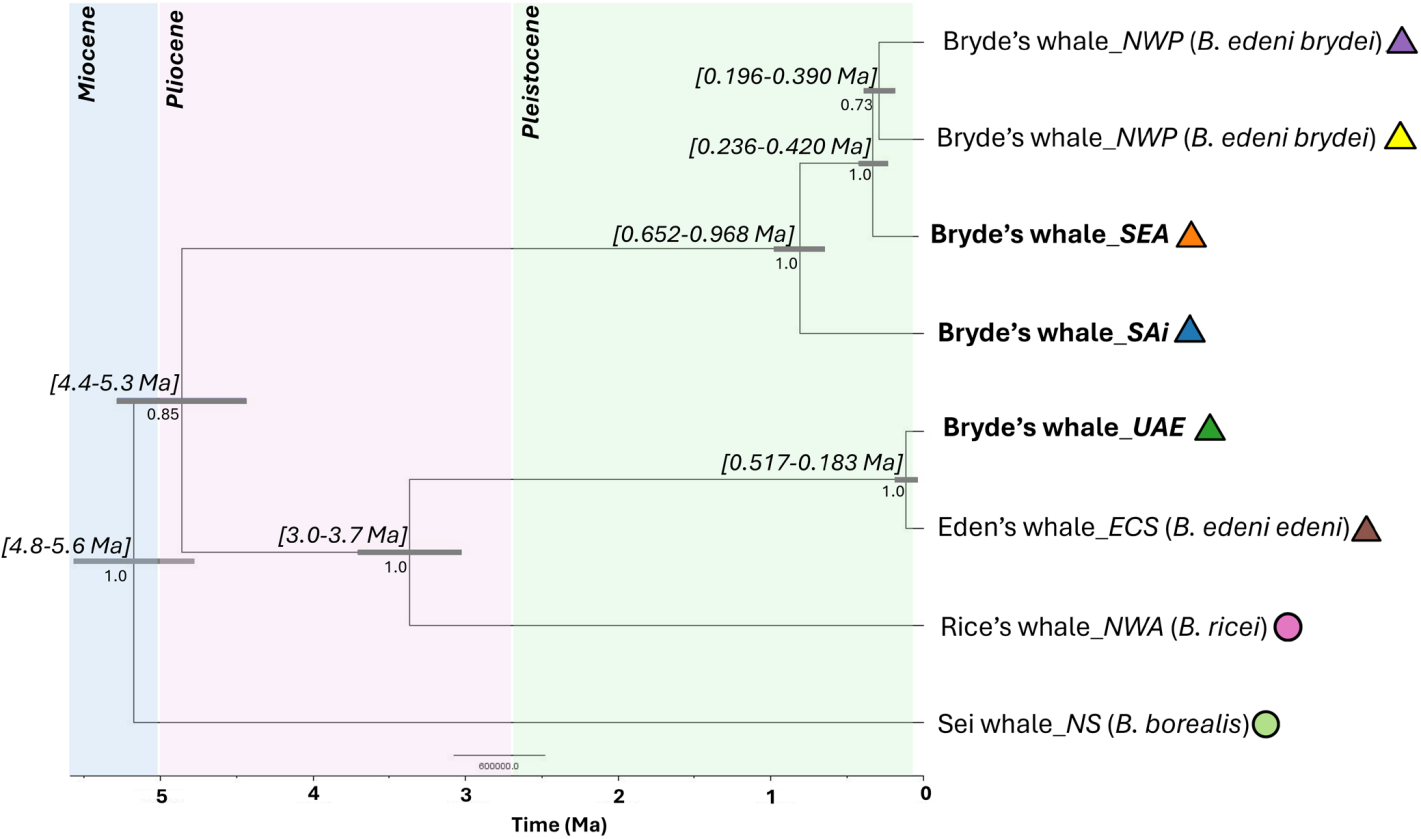
Time‐calibrated gene tree of Bryde's, Eden's, and Rice's whale (with the sei whale as an outgroup) based on BEAST analysis of mitogenome data. Node bars are shown in grey, displaying the 95% CI on each split with the posterior probability indicated below. The upper and lower limits of the divergence time of nodes are indicated in parentheses. The respective epochs are indicated by coloured blocks.

Based on the extreme and significant divergence between species within the balaenopterid dataset, divergence dating was investigated on members of the ‘Bryde's‐sei complex’ to avoid potential skewing of results. Within the Bryde's‐sei dataset, the deepest node on this tree, which indicates the split between the sei whale, Rice's whale and different Bryde's populations, was estimated between 4.8 and 5.6 million years ago (Ma) within the late Miocene/early Pliocene (Figure 4). In this case, Rice's whale, ECS and UAE formed sister taxa with a time to most recent common ancestor (TMRCA) estimated at 3.0–3.7 Ma (Figure 4). In contrast, all other Bryde's whale individuals formed the sister group to this Rice's–ECS–UAE grouping, which shared a most recent common ancestor (MRCA) 4.4–5.3 Ma during the Pliocene. Subsequent diversification of Bryde's whales from the SAi, SEA and NWP then occurred during the Pleistocene, with TMRCA between SAi and the remainder of the Bryde's whales estimated at 654–979 Ka (Figure 4).

## 4 Discussion

The present study provides comprehensive evidence of the existence of multiple unique genetic lineages among Bryde's whales with different geographical distributions and across the two recognised subspecies (*Balaenoptera edeni brydei and Balaenoptera edeni edeni*). Notably, SAi consistently grouped with individuals representing an offshore ecotype ( *B. edeni brydei* ) despite it being recognised as an inshore ecotype. However, according to currently accepted nomenclature, SAi should be classified as Eden's whale ( *B. edeni edeni* ), like the inshore representative from Japan (ECS). At the same time, throughout all phylogenetic and divergence analyses, the SEA population consistently clustered with other offshore Bryde's whale representatives ( *B. edeni brydei* ) from the Northwest Pacific (NWP) yet remained distinctly separated from the SAi population. These findings reinforce the clear genetic distinction between the South African inshore (SAi) and Southeast Atlantic offshore (SEA) Bryde's whale populations, highlighting their unique evolutionary histories. When the geographical comparison is expanded to include an individual from the UAE, the genetic distinctiveness of SAi and SEA remains evident. Each southern African population forms its own separate lineage, both of which are more distantly related to the Northern Indian Ocean (UAE) representative, which itself also constitutes a unique lineage. This broader perspective shows the depth of divergence between these populations and emphasises the importance of recognising their evolutionary independence. When put in context of time, the southern African Bryde's whale populations (SAi and SEA) shared a common ancestor approximately 1 million years ago, whereas both lineages shared a common ancestor with Rice's‐ECS ( *B. edeni edeni* )‐UAE whale lineage between 4 and 5 million years ago indicating all Bryde's and ‘Bryde's‐like’ individuals diverged from the same common ancestor.

The divergence patterns and phylogenetic relationships between southern African Bryde's whales are congruent throughout analyses. SAi and SEA consistently form clusters with NWP offshore representatives in the PCA and group as sister taxa to these individuals in phylogenetic trees. Not once does SAi group cluster closely with the inshore representative from Japan (ECS) identified as *B. edeni edeni* (Sasaki et al. 2005). This result was also detected in phylogenetic relationships constructed in previous studies where similar clustering of SAi and SEA with offshore representatives ( *B. edeni brydei* ) was found based on control region data (Luksenburg et al. 2015; Penry et al. 2018). However, the close clustering of SEA and SAi observed in this study does not necessarily indicate their true phylogenetic relationship. Instances of long‐branch attraction may cause individuals to group with others which are most similar in the absence of representative sequences from other ocean regions. In LBA scenarios, the closest relative of an individual in the dataset is unavailable, and therefore that individual is grouped with the most similar individual among all those compared in that analysis (Felsenstein 1978; Philippe et al. 2005). However, the placement does not necessarily reflect the true phylogenetic relationship and to fully clarify this, larger sample sizes and more geographically representative mitogenome data are required.

Broadening the geographical scope to include Bryde's whale populations from SAi, SEA, and the UAE, the evidence of distinct genetic lineages remains. In contrast to the southern African populations, the UAE population consistently groups closer to the inshore representative from Japan ( *B. edeni edeni* ). Phylogenetic trees based on amino acid data of the balaenopterid and Bryde's‐sei complex datasets support this by showing that the UAE sample consistently groups as a sister taxon with the ECS lineage (Figures S2 and S3). However, due to the single individual representation in combination with the lack of supporting evidence from previous genetic studies in the Arabian/Persian Gulf region, we cannot draw definitive conclusions regarding the potential subspecies classification of the UAE population until more genetic evidence becomes available.

Divergence dating results indicate that the diversification events between Bryde's whale populations assessed in this study occurred during the Pleistocene epoch (2.6 Ma to 11.7 Ka). Nonetheless, all Bryde's whale populations share a MRCA (4.4–5.3 Ma) with Rice's whale and Eden's whale and therefore have the same evolutionary origin. The subsequent divergence events between the Bryde's, Eden's and Rice's lineages correspond with significant changes in sea levels during the Pleistocene (and earlier glacial periods), a period marked by global sea levels approximately 130 m below current levels (Grant et al. 2014). Such changes, particularly during the Last Glacial Maximum (26.5–19 Ka), may have driven the divergence of Bryde's whale populations between major ocean basins, leading to the emergence of distinct genetic lineages across global populations. In addition, previous studies estimating the divergence times of balaenopterids (Sasaki et al. 2005, 2006) have shown similar timeframes, particularly for the divergence date between *B. edeni edeni* and *B. edeni brydei* , which occurred approximately 5.3 Ma (see Figure 4). The distinct separation and phylogenetic relationships between the two currently recognised Bryde's whale subspecies ( *B. edeni edeni* and *B. edeni brydei* ), as demonstrated in this study, were also observed by Kershaw et al. (2013) in a comparison that included a broader range of geographic populations than those used in this study. In more recent work on Eden's whale ( *B. edeni edeni* ), using nuclear genome data, the clear split between subspecies was illustrated again and the TMRCA for Eden's and Bryde's whales was estimated between 5.7 and 9.9 Ma (Lin et al. 2025). This divergence time overlaps slightly with the TMRCA of Bryde's, Eden's, and Rice's whales estimated in this study (4.4–5.3 Ma); however, the difference can be accounted for by Lin et al. (2025) deep fossil‐calibration times and broad phylogenomic dataset. The divergence dates (1 Ma to 190 Ka) between lineages of Bryde's whales align with the accumulation of mutations and differences over many generations. Given the estimated mutation rate for balaenopterid mitogenomes are slow in comparison to other mammalian groups (Jackson et al. 2009), in combination with other life history traits such as long generation times for large cetaceans (Taylor et al. 2007), it is expected that it would take hundreds of thousands to millions of years for clear genetic differentiation to become evident.

Altogether, the evidence presented in this study suggests that there are multiple distinct lineages of Bryde's whales across the Atlantic and Indian Ocean basins, while also highlighting the gaps in sampling and the availability of mitogenome sequences that are representative of all Bryde's whale populations globally. Nonetheless, the phylogenetic trees presented in this study illustrate the overall relationships and groupings between Bryde's whales and other, more divergent balaenopterid species. Furthermore, this study has provided valuable and comprehensive new information about the two key Bryde's whale populations, the SEA and Sai, both central to the species' taxonomic history. Strong bootstrap support and consistent evidence of genetic separation indicate that these two populations should be considered, at the very least, distinct conservation units (Archer et al. 2019; Bachmann et al. 2021). The consistent grouping of the coastally distributed SAi individual with representative offshore Bryde's whales ( *B. edeni brydei* ) challenges the default classification of coastal (inshore) populations as the subspecies *B. edeni edeni* . By using the more informative complete mitogenome, this study supports and builds on previous findings from Penry et al. (2018), and further elucidates the phylogenetic relationships and evolutionary histories of Bryde's whales, a key research priority identified by Kershaw et al. (2013) and Constantine et al. (2018). Overall, this study contributes valuable evidence to improving the overall broader taxonomic understanding of the Bryde's whale while highlighting the useful information that can be gained from analysing comprehensive mitogenomic data in even a few samples. This approach is particularly relevant in investigations of elusive cetacean species where the challenges of small sample size and unresolved taxonomies are prominent.

## 5 Conclusion

This study demonstrates the value of mitochondrial genome analysis to studying elusive cetacean species showing that, even with limited sample sizes, these data can reveal cryptic diversity and identify distinct genetic lineages, directly informing conservation priorities. The occurrence of multiple genetic lineages within cetacean species spanning different ocean basins is common, driving revisions of the number of listed subspecies and species as data become available (Morin et al. 2010; Archer et al. 2019; Leslie et al. 2019; Van Cise et al. 2019). Bryde's whales epitomise the challenges of an elusive species and require urgent, fine‐scale taxonomic investigation at a global scale, given the mounting evidence of cryptic diversity among other baleen whales. Our findings strongly support the taxonomic reassessment of at least the SAi at both regional and global scales. While some taxonomic uncertainties remain, these should not preclude its recognition as a discrete conservation unit, nor delay consideration of its conservation status at a global level. Such recognition would more accurately improve our understanding of its extinction risk and provide a stronger scientific basis for targeted conservation action. A distinct listing would also unlock access to conservation funding streams that rely on Red List categorisation, facilitate integration of the population into international conservation frameworks, and raise the profile of its vulnerability among policymakers and the public. In this way, a separate assessment would not only improve the accuracy of global conservation evaluations but also generate tangible conservation and management benefits for this isolated population. Furthermore, this study re‐emphasised the need for more representative samples from Bryde's whale populations across their global range. Establishing broad, international collaborations and data‐sharing networks is strongly recommended to facilitate the incorporation of nuclear genomic data and integration of ecological information from drone and satellite tagging research. Such coordinated efforts will enable a thorough assessment of genetically distinct populations in conservation planning. These recommendations apply not only to Bryde's whales but also to other cetaceans facing similar challenges. Only through collective action can cryptic genetic diversity in elusive, poorly studied species be resolved, advancing taxonomy, strengthening conservation, and guiding informed management decisions.

## Author Contributions

**Dominique Paynee:** conceptualization (equal), data curation (lead), formal analysis (lead), investigation (equal), writing – original draft (lead), writing – review and editing (equal). **Paulette Bloomer:** conceptualization (equal), formal analysis (supporting), funding acquisition (equal), investigation (supporting), supervision (equal), writing – original draft (equal), writing – review and editing (equal). **Gwenith Penry:** conceptualization (equal), investigation (supporting), supervision (equal), writing – original draft (equal), writing – review and editing (equal). **Simon Elwen:** resources (equal), writing – review and editing (supporting). **Ada Natoli:** resources (equal), writing – original draft (supporting), writing – review and editing (equal). **Vaisakh Dhanalakshmy:** resources (equal), writing – review and editing (supporting). **Thyago Cardoso:** funding acquisition (equal), resources (equal), writing – review and editing (supporting). **Els Vermeulen:** conceptualization (equal), funding acquisition (lead), investigation (supporting), resources (equal), supervision (lead), writing – original draft (equal), writing – review and editing (equal).

## Funding

This work was supported by IUCN Kate Sanderson Grant.

## Disclosure

Benefit‐sharing statement: The data produced in this study provides a baseline database which can be built upon for the Bryde's whale species once more representative global samples become available. Furthermore, the application of mitogenomic data in this study, despite the low sample size, emphasises the utility of such data in the detection of unique genetic lineages, which can be used and applied to other understudied cetaceans. This work also facilitated the collaboration and sharing of genomic data for the overall benefit of the species and highlights how collaboration and flexibility in data sharing can assist in advancing research for elusive species effectively.

## Conflicts of Interest

The authors declare no conflicts of interest.

## Supporting information


**Figure S1:** A Bayesian inference phylogenetic tree generated by MrBayes of the balaenopterid dataset based on full mitogenome data and 1 million MCMCs. The posterior probabilities are indicated at each node.
**Figure S2:** Maximum likelihood tree of the balaenopterid dataset based on the amino acid sequences of 13 mitochondrial protein‐coding genes computed with 10,000 bootstrap replicates. The maximum likelihood bootstrap support is indicated by numbers along branches and at nodes.
**Figure S3:** Maximum likelihood tree of the Bryde's‐Rice's dataset based on the amino acid sequences of 13 mitochondrial protein‐coding genes computed with 10,000 bootstrap replicates. The maximum likelihood bootstrap support is indicated by numbers along branches and at nodes.

## Data Availability

The mitogenomic data of additional balaenopterid species and Bryde's whales from Japan are accessible from GenBank under the listed accession numbers provided in Table 1. Newly generated mitogenomes from the South African inshore population (SAi), the Southeast Atlantic population (SEA), and the United Arab Emirates population (UAE) are publicly available on GenBank under accession numbers provided.

## References

[ece373614-bib-0001] Anderson, J. 1878. “Comprising and Account of the Zoological Results of the Two Expeditions to Western Yunnan 1868 and 1875.” Anatomical and Zoological Researches 1: 551–564.

[ece373614-bib-0002] Archer, F. I. , R. L. Brownell Jr. , B. L. Hancock‐Hanser , et al. 2019. “Revision of Fin Whale *Balaenoptera physalus* (Linnaeus, 1758) Subspecies Using Genetics.” Journal of Mammalogy 100, no. 5: 1653–1670. 10.1093/jmammal/gyz121.

[ece373614-bib-0003] Bachmann, L. , A. A. Cabrera , M. P. Heide‐Jørgensen , et al. 2021. “Mitogenomics and the Genetic Differentiation of Contemporary *Balaena mysticetus* (Cetacea) From Svalbard.” Zoological Journal of the Linnean Society 191, no. 4: 1192–1203. 10.1093/zoolinnean/zlaa082.

[ece373614-bib-0004] Best, P. B. 1977. “Two Allopatric Forms of Bryde's Whale Off South Africa.” Report International Whaling Commission 1: 10–38.

[ece373614-bib-0005] Best, P. B. 2001. “Distribution and Population Separation of Bryde's Whale *Balaenoptera edeni* Off Southern Africa.” Marine Ecology Progress Series 220: 277–289.

[ece373614-bib-0006] Bouckaert, R. , J. Heled , D. Kühnert , et al. 2014. “BEAST 2: A Software Platform for Bayesian Evolutionary Analysis.” PLoS Computational Biology 10, no. 4: 1003537. 10.1371/journal.pcbi.1003537. PMC3985171 24722319

[ece373614-bib-0008] Camacho, C. , G. Coulouris , V. Avagyan , et al. 2009. “BLAST+: Architecture and Applications.” BMC Bioinformatics 10, no. 1: 421. 10.1186/1471-2105-10-421. 20003500 PMC2803857

[ece373614-bib-0009] Chang, C. C. , C. C. Chow , L. C. A. M. Tellier , S. Vattikuti , S. M. Purcell , and J. J. Lee . 2015. “Second‐Generation PLINK: Rising to the Challenge of Larger and Richer Datasets.” GigaScience 4: 7. 10.1186/s13742-015-0047-8. 25722852 PMC4342193

[ece373614-bib-0010] Committee on Taxonomy . 2025. List of Marine Mammal Species and Subspecies. Society for Marine Mammalogy. https://marinemammalscience.org/science‐and‐publications/list‐marine‐mammal‐species‐subspecies/.

[ece373614-bib-0011] Constantine, R. , T. Iwata , S. L. Nieukirk , and G. S. Penry . 2018. “Future Directions in Research on Bryde's Whales.” Frontiers in Marine Science 5: 33. 10.3389/fmars.2018.00333.

[ece373614-bib-0012] Dalpaz, L. , A. L. Cypriano‐Souza , L. Lodi , L. Wedekin , and F. Daura‐Jorge . 2023. “Bryde's Whales in South Brazil Bight: Evidence of Low Genetic Diversity and Seasonal Habitat Use.” Marine Biology 170, no. 8: 94. 10.1007/s00227-023-04241-0.

[ece373614-bib-0013] De Coster, W. , and R. Rademakers . 2023. “NanoPack2: Population‐Scale Evaluation of Long‐Read Sequencing Data.” Bioinformatics 39, no. 5: 311. 10.1093/bioinformatics/btad311. PMC10196664 37171891

[ece373614-bib-0055] Donath, A. , F. Jühling , M. Al‐Arab , et al. 2019. “Improved Annotation of Protein‐Coding Genes Boundaries in Metazoan Mitochondrial Genomes.” Nucleic Acids Research 47, no. 20: 10543–10552. 10.1093/nar/gkz833. 31584075 PMC6847864

[ece373614-bib-0014] Felsenstein, J. 1978. “Cases in Which Parsimony or Compatibility Methods Will Be Positively Misleading.” Systematic Zoology 27, no. 4: 401–410. 10.2307/2412923.

[ece373614-bib-0015] Grant, K. M. , E. J. Rohling , C. B. Ramsey , et al. 2014. “Sea‐Level Variability Over Five Glacial Cycles.” Nature Communications 5, no. 1: 5076. 10.1038/ncomms6076. 25254503

[ece373614-bib-0016] Gurevich, A. , V. Saveliev , N. Vyahhi , and G. Tesler . 2013. “QUAST: Quality Assessment Tool for Genome Assemblies.” Bioinformatics (Oxford, England) 29, no. 8: 1072–1075. 10.1093/bioinformatics/btt086. 23422339 PMC3624806

[ece373614-bib-0017] Helfrich, P. , E. Rieb , G. Abrami , A. Lücking , and A. Mehler . 2018. “TreeAnnotator: Versatile Visual Annotation of Hierarchical Text Relations.” In Proceedings of the Eleventh International Conference on Language Resources and Evaluation (LREC 2018). European Language Resources Association (ELRA).

[ece373614-bib-0018] Hohenlohe, P. A. , W. C. Funk , and O. P. Rajora . 2021. “Population Genomics for Wildlife Conservation and Management.” Molecular Ecology 30, no. 1: 62–82. 10.1111/mec.15720. 33145846 PMC7894518

[ece373614-bib-0019] Jackson, J. A. , C. S. Baker , M. Vant , D. J. Steel , L. Medrano‐Gonzalez , and S. R. Palumbi . 2009. “Big and Slow: Phylogenetic Estimates of Molecular Evolution in Baleen Whales (Suborder Mysticeti).” Molecular Biology and Evolution 26, no. 11: 2427–2440. 10.1093/molbev/msp169. 19648466

[ece373614-bib-0020] Junge, G. C. A. 1950. “On a Specimen of the Rare Fin Whale, *Balaenoptera edeni* Anderson, Stranded on Pulu Sugi Near Singapore.” Zoologische Verhandelingen 9, no. 1: 1–26.

[ece373614-bib-0021] Kanda, N. , M. Goto , H. Kato , M. V. McPhee , and L. A. Pastene . 2007. “Population Genetic Structure of Bryde's Whales ( *Balaenoptera brydei* ) at the Inter‐Oceanic and Trans‐Equatorial Levels.” Conservation Genetics 8, no. 4: 853–864. 10.1007/s10592-006-9232-8.

[ece373614-bib-0022] Katoh, K. , J. Rozewicki , and K. D. Yamada . 2019. “MAFFT Online Service: Multiple Sequence Alignment, Interactive Sequence Choice and Visualization.” Briefings in Bioinformatics 20, no. 4: 1160–1166. 10.1093/bib/bbx108. 28968734 PMC6781576

[ece373614-bib-0023] Kershaw, F. , M. S. Leslie , T. Collins , et al. 2013. “Population Differentiation of 2 Forms of Bryde's Whales in the Indian and Pacific Oceans.” Journal of Heredity 104, no. 6: 755–764. 10.1093/jhered/est057. 24081988

[ece373614-bib-0024] Kolmogorov, M. , J. Yuan , Y. Lin , and P. A. Pevzner . 2019. “Assembly of Long, Error‐Prone Reads Using Repeat Graphs.” Nature Biotechnology 37, no. 5: 540–546. 10.1038/s41587-019-0072-8. 30936562

[ece373614-bib-0026] Leslie, M. S. , F. I. Archer , and P. A. Morin . 2019. “Mitogenomic Differentiation in Spinner ( *Stenella longirostris* ) and Pantropical Spotted Dolphins ( *S. attenuata* ) From the Eastern Tropical Pacific Ocean.” Marine Mammal Science 35, no. 2: 522–551. 10.1111/mms.12545.

[ece373614-bib-0027] Letunic, I. , and P. Bork . 2021. “Interactive Tree of Life (iTOL) v5: An Online Tool for Phylogenetic Tree Display and Annotation.” Nucleic Acids Research 49, no. W1: W293–W296. 10.1093/nar/gkab301. 33885785 PMC8265157

[ece373614-bib-0028] Li, H. 2018. “Minimap2: Pairwise Alignment for Nucleotide Sequences.” Bioinformatics 34, no. 18: 3094–3100. 10.1093/bioinformatics/bty191. 29750242 PMC6137996

[ece373614-bib-0029] Li, H. , B. Handsaker , A. Wysoker , et al. 2009. “The Sequence Alignment/Map Format and SAMtools.” Bioinformatics (Oxford, England) 25, no. 16: 2078–2079. 10.1093/bioinformatics/btp352. 19505943 PMC2723002

[ece373614-bib-0030] Lin, Y.‐T. , F. Hui , W. Han , et al. 2025. “Chromosome‐Level Genome Assembly of Eden's Whale Clarifies the Taxonomy and Speciation of Bryde's Whale Complex.” Molecular Biology and Evolution 42, no. 10: msaf234. 10.1093/molbev/msaf234. 40973465 PMC12492004

[ece373614-bib-0031] Luksenburg, J. A. , A. Henriquez , and G. Sangster . 2015. “Molecular and Morphological Evidence for the Subspecific Identity of Bryde's Whales in the Southern Caribbean.” Marine Mammal Science 31, no. 4: 1568–1579. 10.1111/mms.12236.

[ece373614-bib-0032] Minh, B. Q. , H. A. Schmidt , O. Chernomor , et al. 2020. “IQ‐TREE 2: New Models and Efficient Methods for Phylogenetic Inference in the Genomic Era.” Molecular Biology and Evolution 37, no. 5: 1530–1534. 10.1093/molbev/msaa015. 32011700 PMC7182206

[ece373614-bib-0033] Morin, P. A. , F. I. Archer , A. D. Foote , et al. 2010. “Complete Mitochondrial Genome Phylogeographic Analysis of Killer Whales ( *Orcinus orca* ) Indicates Multiple Species.” Genome Research 20, no. 7: 908–916. 20413674 10.1101/gr.102954.109 PMC2892092

[ece373614-bib-0034] Morin, P. A. , K. K. Martien , A. R. Lang , et al. 2023. “Guidelines and Quantitative Standards for Improved Cetacean Taxonomy Using Full Mitochondrial Genomes.” Journal of Heredity 114, no. 6: 612–624. 10.1093/jhered/esad049. 37647537

[ece373614-bib-0035] Olsen, O. 1913. “On the External Characters and Biology of Bryde's Whale *Balaenoptera brydei* , a New Rorqual From the Coast of South Africa.” Proceedings of the Zoological Society of London 83, no. 4: 1073–1090. 10.1111/j.1096-3642.1913.tb02005.x.

[ece373614-bib-0036] Paynee, D. , E. Vermeulen , G. Penry , et al. 2026. “Low Genetic Diversity and Regional Isolation of South Africa's Inshore Bryde's Whales.” Conservation Genetics 27, no. 2: 26. 10.1007/s10592-025-01749-4.

[ece373614-bib-0037] Penry, G. , P. S. Hammond , V. G. Cockcroft , P. B. Best , M. Thornton , and J. A. Graves . 2018. “Phylogenetic Relationships in Southern African Bryde's Whales Inferred From Mitochondrial DNA: Further Support for Subspecies Delineation Between the Two Allopatric Populations.” Conservation Genetics 19, no. 6: 1349–1365. 10.1007/s10592-018-1105-4.

[ece373614-bib-0038] Philippe, H. , Y. Zhou , H. Brinkmann , N. Rodrigue , and F. Delsuc . 2005. “Heterotachy and Long‐Branch Attraction in Phylogenetics.” BMC Evolutionary Biology 5: 50. 10.1186/1471-2148-5-50. 16209710 PMC1274308

[ece373614-bib-0039] Rambaut, A. 2018. Figtree Ver 1.4.4. Institute of Evolutionary Biology, University of Edinburgh.

[ece373614-bib-0040] Rambaut, A. , A. J. Drummond , D. Xie , G. Baele , and M. A. Suchard . 2018. “Posterior Summarization in Bayesian Phylogenetics Using Tracer 1.7.” Systematic Biology 67, no. 5: 901–904. 10.1093/sysbio/syy032. 29718447 PMC6101584

[ece373614-bib-0041] RCore team . 2025. RStudio: Integrated Development Environment for R. Posit Software. http://www.posit.co/.

[ece373614-bib-0042] Romiguier, J. , V. Ranwez , E. J. Douzery , and N. Galtier . 2010. “Contrasting GC‐Content Dynamics Across 33 Mammalian Genomes: Relationship With Life‐History Traits and Chromosome Sizes.” Genome Research 20, no. 8: 1001–1009. 10.1101/gr.104372.109. 20530252 PMC2909565

[ece373614-bib-0043] Ronquist, F. , M. Teslenko , P. van der Mark , et al. 2012. “MrBayes 3.2: Efficient Bayesian Phylogenetic Inference and Model Choice Across a Large Model Space.” Systematic Biology 61, no. 3: 539–542. 10.1093/sysbio/sys029. 22357727 PMC3329765

[ece373614-bib-0044] Rosel, P. , P. Corkeron , and M. Soldevilla . 2022. “*Balaenoptera ricei*. The IUCN Red List of Threatened Species 2022.”

[ece373614-bib-0045] Rosel, P. E. , L. A. Wilcox , T. K. Yamada , and K. D. Mullin . 2021. “A New Species of Baleen Whale (Balaenoptera) From the Gulf of Mexico, With a Review of Its Geographic Distribution.” Marine Mammal Science 37, no. 2: 577–610. 10.1111/mms.12776.

[ece373614-bib-0046] Sasaki, T. , M. Nikaido , H. Hamilton , et al. 2005. “Mitochondrial Phylogenetics and Evolution of Mysticete Whales.” Systematic Biology 54, no. 1: 77–90. 10.1080/10635150590905939. 15805012

[ece373614-bib-0047] Sasaki, T. , M. Nikaido , S. Wada , et al. 2006. “ *Balaenoptera omurai* Is a Newly Discovered Baleen Whale That Represents an Ancient Evolutionary Lineage.” Molecular Phylogenetics and Evolution 41, no. 1: 40–52. 10.1016/j.ympev.2006.03.032. 16843687

[ece373614-bib-0048] Tamura, K. , G. Stecher , and S. Kumar . 2021. “MEGA11: Molecular Evolutionary Genetics Analysis Version 11.” Molecular Biology and Evolution 38, no. 7: 3022–3027. 10.1093/molbev/msab120. 33892491 PMC8233496

[ece373614-bib-0049] Taylor, B. , S. J. Chivers , J. Larese , and W. F. Perrin . 2007. Generation Length and Percent Mature Estimates for IUCN Assessments of Cetaceans. NOAA, NMFS, Southwest Fisheries Science Center Administrative Report LJ‐07‐01 [Preprint].

[ece373614-bib-0050] Taylor, B. L. , F. I. Archer , K. K. Martien , et al. 2017. “Guidelines and Quantitative Standards to Improve Consistency in Cetacean Subspecies and Species Delimitation Relying on Molecular Genetic Data.” Marine Mammal Science 33, no. S1: 132–155. 10.1111/mms.12411.

[ece373614-bib-0051] Taylor, B. L. , W. F. Perrin , R. R. Reeves , et al. 2017. “Why We Should Develop Guidelines and Quantitative Standards for Using Genetic Data to Delimit Subspecies for Data‐Poor Organisms Like Cetaceans.” Marine Mammal Science 33, no. S1: 12–26. 10.1111/mms.12413.

[ece373614-bib-0052] Vachon, F. , H. Whitehead , and T. R. Frasier . 2018. “What Factors Shape Genetic Diversity in Cetaceans?” Ecology and Evolution 8, no. 3: 1554–1572. 10.1002/ece3.3727. 29435232 PMC5792597

[ece373614-bib-0053] Van Cise, A. M. , R. W. Baird , C. S. Baker , et al. 2019. “Oceanographic Barriers, Divergence, and Admixture: Phylogeography and Taxonomy of Two Putative Subspecies of Short‐Finned Pilot Whale.” Molecular Ecology 28, no. 11: 2886–2902. 10.1111/mec.15107. 31002212

[ece373614-bib-0054] Vaser, R. , I. Sović , N. Nagarajan , and M. Šikić . 2017. “Fast and Accurate de Novo Genome Assembly From Long Uncorrected Reads.” Genome Research 27, no. 5: 737–746. 10.1101/gr.214270.116. 28100585 PMC5411768

