# Supplementary material for: Unravelling Taxonomic Complexity in Elusive Cetaceans: Mitogenome Insights into Evolutionary History and Cryptic Diversity of Bryde's Whales
Source: Ecol Evol. 2026 May 3;16(5):e73614. doi: 10.1002/ece3.73614 (PMC13135864; doi:10.1002/ece3.73614)
Supplement: Supplementary file 1 — Figure S1: A Bayesian inference phylogenetic tree generated by MrBayes of the balaenopterid dataset based on full mitogenome data and 1 million MCMCs. The posterior probabilities are indicated at each node. Figure S2: Maximum likelihood tree of the balaenopterid dataset based on the amino acid sequences of 13 mitochondrial protein‐coding genes computed with 10,000 bootstrap replicates. The maximum likelihood bootstrap support is indicated by numbers along branches and at nodes. Figure S3: Maximum likelihood tree of the Bryde's‐Rice's dataset based on the amino acid sequences of 13 mitochondrial protein‐coding genes computed with 10,000 bootstrap replicates. The maximum likelihood bootstrap support is indicated by numbers along branches and at nodes. [file ECE3-16-e73614-s001.docx]

**Supplementary Materials**


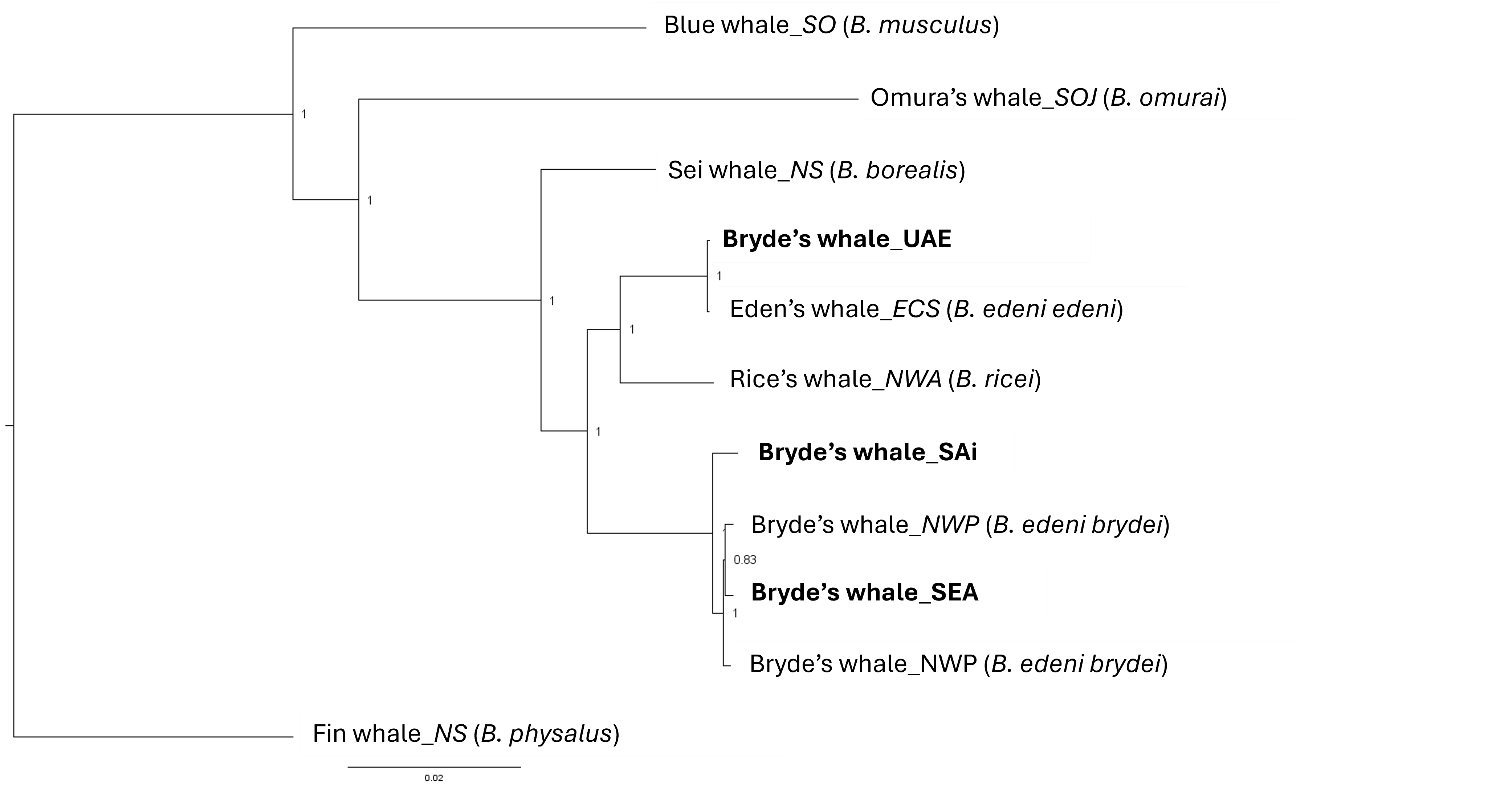


**Figure 1.** A Bayesian inference phylogenetic tree generated by MrBayes of the balaenopterid dataset based on full mitogenome data and 1 million MCMCs. The posterior probabilities are indicated at each node.


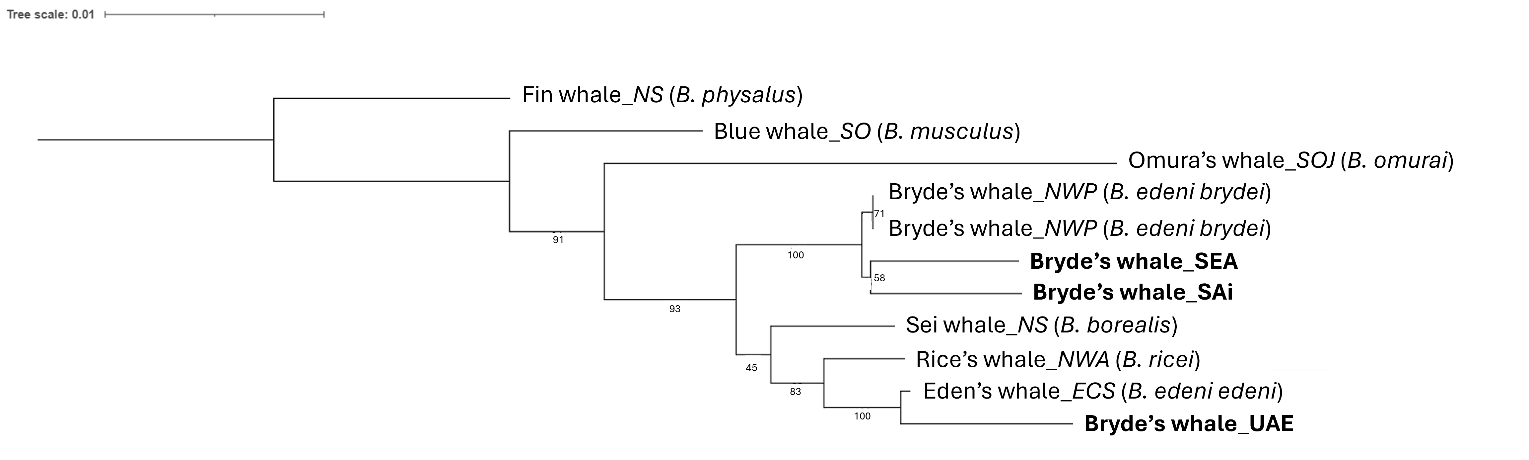


**Figure 2.** Maximum likelihood tree of the balaenopterid dataset based on the amino acid sequences of 13 mitochondrial protein-coding genes computed with 10,000 bootstrap replicates. The maximum likelihood bootstrap support is indicated by numbers along branches and at nodes.


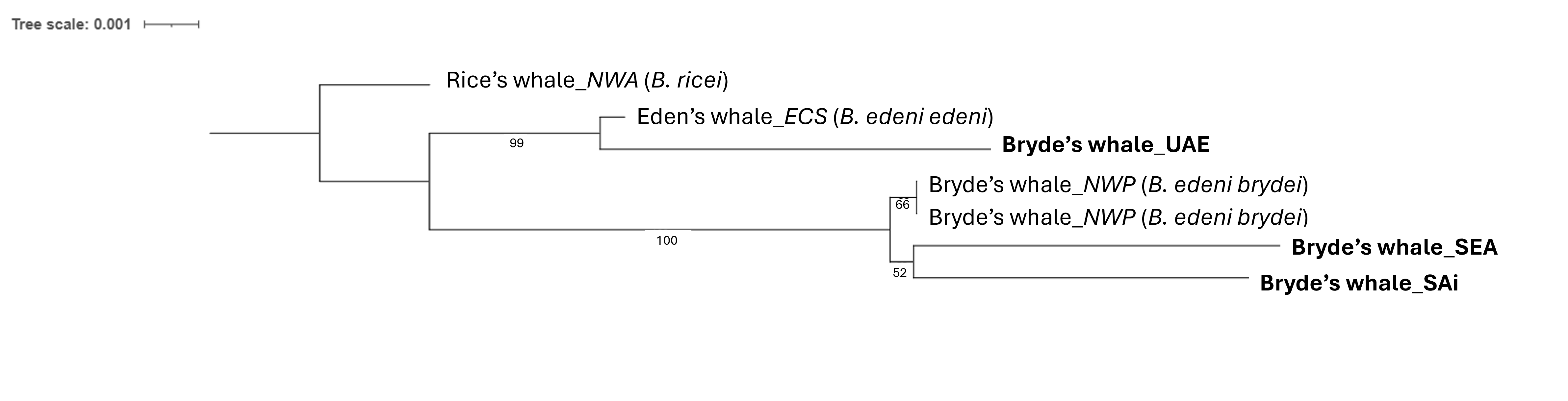


**Figure 3**. Maximum likelihood tree of the Bryde’s-Rice’s dataset based on the amino acid sequences of 13 mitochondrial protein-coding genes computed with 10,000 bootstrap replicates. The maximum likelihood bootstrap support is indicated by numbers along branches and at nodes.
